# Prevalence of probable eating disorders and associated risk factors in children and adolescents aged 5–16 years in Al Ain City, United Arab Emirates: observational case–control study

**DOI:** 10.1186/s40337-023-00840-w

**Published:** 2023-07-07

**Authors:** Naji Al Mestaka, Amna Alneyadi, Ali AlAhbabi, Abdulla AlMatrushi, Rehab AlSaadi, Latifa Baynouna Alketbi

**Affiliations:** Department of Family Medicine, Ambulatory Healthcare Services, Abu Dhabi Health Services Company, Al Ain, United Arab Emirates

**Keywords:** Eating disorders, Childhood obesity, Depression, Screening, SCOFF questionnaire

## Abstract

**Background:**

Eating disorders, including anorexia, bulimia, and binge eating, have become a significant health concern among young children and adolescents worldwide. The objective of this study is to examine the probable eating disorder prevalence and associated risk factors among obese and normal-weight children and adolescents aged (5–16 years) in Al Ain, United Arab Emirates (UAE).

**Methods:**

This observational case–control study utilized data obtained from electronic medical records (age, gender, body measurements). SCOFF questionnaire and Patient Health Questionnaire-2 (PHQ-2) were used to estimate the probable prevalence of eating disorders and depression, respectively, in children and adolescents. The study was conducted in Al Ain Ambulatory health services clinics from 2018 to 2019. Descriptive statistics and linear regression analysis were employed for data analysis.

**Results:**

A total of 551 subjects participated in the study, with 288 (52%) categorized as normal-weight and 263 (48%) as obese. Among the obese participants, there was an equal distribution of males and females. Screening for eating disorders using the SCOFF questionnaire revealed that approximately 42% of the obese participants had a positive SCOFF result, indicating abnormal eating behaviors. In contrast, only 7% of the normal-weight participants had a positive SCOFF result. A significant positive correlation was observed between a positive SCOFF screening result, PHQ-2 score, and the participants' weight at the age of 6 years.

**Conclusion:**

This study represents the first attempt to assess the probable prevalence of the risk of eating disorders in children and adolescents in the UAE. This young population have high risk of eating disorders and it was significantly higher in obese children than normal weight children. These results highlight the importance of addressing eating disorders in this population and the need for early detection and intervention strategies.

## Key messages

- Eating disorders are a growing problem worldwide.
- There is a lack of screening for eating disorders in children and adolescents.
- SCOFF is a simple, easy to use screening tool for screening eating disorders.
- Eating disorders in children and adolescents can be linked to depression and early childhood obesity.
- There is an association between obesity and eating disorders in children and adolescents.

## Abstract (plain language summary)

In the United Arab Emirates (UAE), little research has been done to study children with a risk of developing abnormal eating behaviors, especially in school-aged children and adolescents with increased weight. In this research, a questionnaire was collected from children and adolescents, asking them specific questions used to screen for abnormal eating behaviors in this age group. Based on the screening questions used in this study, around 23% of the children and adolescents probably have abnormal eating behaviors, especially those with increased weight. This study will help us to create community services that aim for the early screening of school children for abnormal eating behaviors to provide treatment and intervention.

## Background

Childhood and adolescent obesity pose a significant health challenge that jeopardizes the well-being of future generations. The prevalence of obesity in children has been steadily increasing since 1988 [1]. According to the Centers for Disease Control (CDC), in the period between 2017 and 2018, approximately 13.4% of children aged 2–5 years, around 20.3% of those aged 6–11 years, and approximately 21.2% of adolescents aged 12–19 years were obese [2]. The association between psychiatric disorders and childhood obesity is complex; it remains unclear whether conditions like depression or eating disorders are the cause or consequence of obesity. Nevertheless, overweight and obese children and adolescents are more susceptible to experience psychiatric disorders such as depression and eating disorders compared to their peers of normal weight [3]. In the United Arab Emirates (UAE), childhood obesity is a prominent issue, prompting studies to quantify the extent of obesity among children and adolescents. Research conducted on school-aged children revealed a prevalence of overweight and obesity of approximately 28.2% [4]. Similarly, among adolescents, another study reported a prevalence of obesity of around 34.7% [5]. Regrettably, there have been no studies focusing on the link between childhood obesity and mental health disorders in the UAE. Therefore, the aim of this study is to estimate the likely prevalence of mental health disorders, such as eating disorders, among obese children and adolescents aged 5–16 years attending primary health care centers in Al Ain over a two-year period (2018–2019).

## Methods

This observational study was conducted as part of a larger prospective cross-sectional study involving obese children aged between 5 and 16 years old. The research took place et al. Ain Ambulatory Health Services Clinics from 2018 and 2019. To classify children as obese or normal-weight, we utilized the World Health Organization (WHO) body mass index (BMI) z-score reference. obesity was defined as a BMI z-score of > + 2 standard deviations (equivalent to BMI 30 kg/m^2^ at 19 years), while overweight was defined as a BMI z-score of > + 1 standard deviation (equivalent to BMI 25 kg/m^2^ at 19 years) [6].

The study comprised a total of 551 participants aged between 5 and 16 years. Participants were recruited from two distinct sources. The first source involved retrospective recruitment from the database of Abu Dhabi Health Authority's (SEHA) school screening program in Al Ain region during the period of 2018–2019. Subjects for the second source were recruited prospectively as walk-in patients from various ambulatory health care services centers. To ensure the study's focus on a specific population, we excluded from our study certain subjects based on certain predefined criteria. Those excluded from the study were non-nationals, children below the age of five or above 16 years, individuals classified as overweight or underweight according to the WHO definition, and children who were obese due to other medical conditions. To ensure representation from diverse areas, healthcare centers were chosen by simple randomization. This selection process encompassed all healthcare centers in Al Ain city, including both rural and urban healthcare centers.

The data collection and measurement tools utilized in this study included the following:

1. Self-report questionnaire: Participants or their parents were asked to complete a questionnaire that gathered demographic information and included questions related to lifestyle factors. The questionnaire was administered either through face-to-face interviews or telephone interviews following the obtaining of consent.
2. Anthropometric indices: Age, gender, weight, height, blood pressure, and birth weight data were collected from the participants' medical records. These measurements were used to assess various anthropometric indices relevant to the study.
3. Questionnaires on determinants of obesity, depression, and eating disorders: To investigate the factors influencing obesity in children and its association with depression and eating disorders, specific questionnaires were administered. These questionnaires were designed to gather information related to these areas of interest.

By employing a combination of self-report questionnaires, medical record data, and targeted questionnaires, the study aimed to comprehensively assess the determinants of childhood obesity while exploring its relationship with depression and eating disorders.

### Patient health questionnaire-2 (PHQ-2)

The PHQ-2 is a concise depression screening tool, mainly asking two simple questions about mood and anhedonia. It serves as a shorter version of the PHQ-9, which comprises a more extensive set of questions. The PHQ-2 has been found to exhibit 97% sensitivity and 67% specificity [7].

### SCOFF questionnaire

In primary care settings, various screening questionnaires are available for assessing eating disorders, including the Eating Disorder Screen for Primary Care (EDS-PC), the Screen for Disordered Eating, and the SCOFF questionnaire [8].

SCOFF is a screening tool for eating disorders (bulimia nervosa and anorexia nervosa). It was first developed in 1999 by Morgan et al. [9] and validated in 2002 as a screening tool for eating disorders in primary care [10]. It consists of five simple questions concerning aspects such as related to sickness, control overweight (one stone or 6.5 kg), feeling of fatness, and food. With two positive answers or more, the SCOFF questionnaire exhibits a sensitivity of 86% and specificity of 83% [11].

### Statistical analysis

Statistical analysis was performed using the SPSS statistics software, version 19 IBM Corp., Armonk, NY, USA. Linear regression analysis was used to determine any association between obesity and the PHQ-2 and SCOFF results. The results were presented as odds ratios (ORs) with 95% confidence intervals (CIs). Statistical significance was indicated by a *p*-value < 0.05.

## Results

The study included a total of 551 participants. Among them, 288 (52%) were classified as normal-weight, while 263 (48%) were classified as obese. Within the group of the obese participants, 46% (277) were females, while 54% (274) were male (Table 1).

**Table 1:** Baseline characteristics of the participants

Category	Normal (%)	Obese (%)	Total
Gender			
Female	156 (54)	121 (46)	277
Male	132 (46)	142 (54)	274
Total	288 (52)	263 (48)	551

Normal weight participants and obese are based on the WHO BMI z score definition, as mentioned earlier

Table 2 present the responses of the participants to the SCOFF questionnaire. The results are as follows:

**Table 2:** SCOFF questionnaire answers

SCOFF questions		Normal	Obese	Total
*1-Do you make yourself sick because you feel uncomfortable?*				
Answers	No	284 (99%)	240 (91%)	524
	Yes	4 (1%)	23 (8%)	27
	Total	288	263	551
*2-Do you worry you have lost control over how much you eat?*				
Answers	No	266 (91%)	163 (62%)	429
	Yes	22 (8%)	100 (38%)	122
	Total	288	263	551
*3-Have you recently lost more than 6.35 kg in the month?*				
Answers	No	286 (99%)	257 (97%)	543
	Yes	2 (< 1%)	6 (2%)	8
	Total	288	263	551
*4-Do you believe yourself to be fat when others say you are thin?*				
Answers	No	264 (91%)	116 (44%)	380
	Yes	24 (8%)	147 (55%)	171
	Total	288	263	551
*5-Would you say food dominates your life?*				
Answers	No	261 (90%)	154 (57%)	415
	Yes	27 (9%)	109 (41%)	136
	Total	288	263	551

For question 1: “Do you make yourself sick because you feel uncomfortable?”.

- Among normal-weight participants, 1% answered yes.
- Among obese participants, 8% answered yes.

For question 2: “Do you worry you have lost control over how much you eat?”.

- Among normal-weight participants, 8% answered yes.
- Among obese participants, 38% answered yes.

For question 3: “Asked whether the participants lost around 6.35 kg or a stone in the last month?”.

- Both groups majority answered no.

For question 4: “Do you believe yourself to be fat when others say you are thin?”.

- Around 55% of obese participants answered yes, indicating that they believe themselves to be fat.
- Approximately 8% of normal-weight participants answered yes to the same question

For question 5: “Would you say food dominates your life?”.

- 41% of obese participants indicated that food dominates their life
- 9% of normal-weight participants expressed the same belief.

These responses provide insight into the participants' perspectives regarding various aspects related to eating disorders and body image.

To specifically screen for eating disorders among the participants, SCOFF questionnaire answers were categorized as follow:

- A score of 0 for those who answered “No” to all SCOFF questions.
- A score of 1 indicated a positive response to one SCOFF question.
- A score of 2 or more was considered a positive screen, indicating that the participants answered “yes” to two or more of the SCOFF questions [9].

Based on these criteria, Table 3 demonstrates the distribution of SCOFF scores among the participants. Among obese participants, 42% had a positive SCOFF result for 2 questions, and 40% had a positive result for 1 question. In comparison, only 7% of normal-weight participants had a positive SCOFF result for 2 questions, and approximately 10% had a positive result for 1 question. Negative screening for eating disorders was observed in 17% of obese children and 84% of normal-weight children.

**Table 3:** The probable prevalence of eating disorders based on the SCOFF questionnaire

SCOFF	Normal	Obese	Total
= 0	241 (84%)	46 (17%)	287
= 1	28 (10%)	107 (40%)	135
≥ 2	19 (7%)	110 (42%)	129
Total	288	263	551

0 = negative, 1 = possible, 2 = positive screening results

Overall, out of the total population of 551 participants, 129 individuals had a positive SCOFF screening, surpassing the recommended cutoff score of 2 or more. This finding suggests that the estimated probable prevalence of eating disorders among children and adolescents up to the age of 16 years was approximately 23.4%. Since SCOFF is a screening tool, these participants received a follow up appointment with pediatrician for further evaluation to confirm the diagnosis of eating disorders, whether they were obese or normal weight participants.

**Fig. 1:**
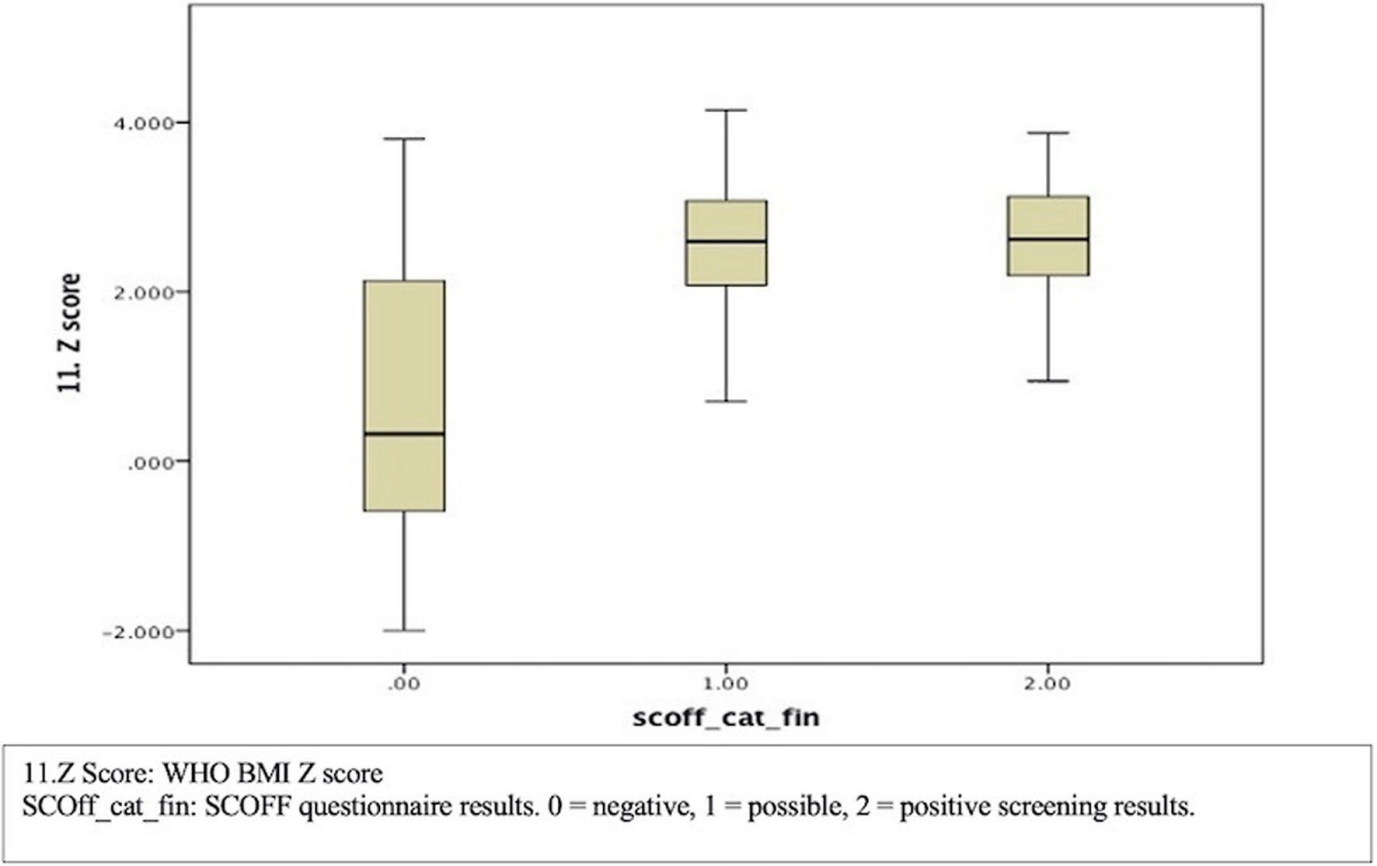
Box plot of the distribution of the SCOFF questionnaire results with the WHO BMI Z score

Figure 1 illustrates the correlation between the distribution of the SCOFF questionnaire results and the WHO BMI Z score of the participants. The graph reveals that a higher proportion of participants with lower Z score values had normal screening results. Conversely, participants with positive screening results were predominantly observed among individuals with higher BMI Z score values, particularly among the obese participants. This suggests a positive association between higher BMI Z scores and an increased likelihood of positive SCOFF screening results, indicating a potential link between higher body mass index and the presence of eating disorder symptoms.

According to the information provided in Table 4, there are significant associations between the SCOFF screening results and two variables: the weight of the participant at six years old (grade 1) and the score of the PHQ-2 questionnaire.

1. Weight at six years old: A significant positive relationship was observed between the SCOFF screening test results and the weight of the participants at six years of age (grade 1). The *p*-value associated with this relationship was found to be less than 0.001, indicating a highly significant association.
2. PHQ-2 score: Similarly, a significant positive relationship was identified between the SCOFF screening results and the scores obtained from the PHQ-2 questionnaire. The *p*-value associated with this relationship was also found to be less than 0.001, indicating a highly significant association.

**Table 4:** Regression of SCOFF determinants

Model	Unstandardized coefficients		Standardized coefficients		
	B	Std. error	Beta	t	Sig
PHQ2	0.372	0.07	0.291	5.307	< 0.001
(Weight at grade 1 or 6 years old)	0.024	0.007	0.183	3.346	< 0.001

These findings suggest that both the weight of the participant at six years old and the score on the PHQ-2 questionnaire have a notable influence on the results of the SCOFF screening test for potential eating disorders.

## Discussion

To the best of our knowledge, this study represents the first of its kind in estimating the probable prevalence of the risk of eating disorders in children and adolescents in the UAE, utilizing the SCOFF questionnaire as a screening tool. The results indicate the possible prevalence of the risk of eating disorders in children and adolescents up to the age of 16 years to be approximately 23.4%.

Comparisons can be made with similar studies conducted worldwide using the SCOFF questionnaire as a screening tool. For instance, a large, nationally representative household survey conducted in Ireland revealed a probable risk of eating disorders in 16.2% of the population using the SCOFF questionnaire as a screening tool [12]. In Austria, a study involving 3610 participants aged 10–18 years showed that 30.9% of girls and 14.6% of boys were at a higher risk of developing eating disorders [13].

In contrast, a nationally representative study in the United States reported a lower prevalence of eating disorders among children and adolescents aged 8–11, with approximately 0.1% based on the DSM-IV diagnostic criteria [14].

These comparisons highlight the variations in eating disorder prevalence across different populations and regions, emphasizing the importance of conducting localized studies to understand the specific prevalence rates and risk factors within a particular population, such as the one conducted in the UAE.

Community studies that utilize dimensional measures, such as SCOFF, have consistently reported a higher prevalence of disordered eating behaviors in youths ranging from 14 to 22%. These rates are notably higher than those found in studies that applied strict DSM-IV diagnostic criteria.

It is essential to consider these differences in prevalence rates when comparing the actual burden of eating disorders among younger children worldwide. The dimensional approach captures a broader spectrum of disordered eating behaviors, including subclinical symptoms and behaviors that may not meet the strict diagnostic criteria for a specific eating disorder. This broader inclusion leads to higher prevalence estimates in community studies.

On the other hand, studies that rely on the DSM-IV criteria for diagnosis tend to have lower prevalence rates since they require meeting specific criteria for a formal diagnosis. These studies may overlook milder or subthreshold cases that still indicate significant disordered eating behaviors.

Despite the difference in defining obesity and eating disorders, numerous studies have been conducted to investigate the relationship between obesity and eating disorders. Among these disorders, binge-eating has been found to be the most prevalent eating disorder associated with obesity [15]. The present study observed that 42% of participants classified as obese, as per the WHO BMI Z score definition also gave a positive response to two or more questions on the SCOFF questionnaire. In contrast, only approximately 7% of normal-weight participants had a positive SCOFF questionnaire result. These findings highlight a higher prevalence of eating disorder symptoms among individuals with obesity.

Indeed, the finding that approximately 7% of normal-weight participants had a positive SCOFF test result highlights an important point. Until recently, there has been growing recognition of a subset of individuals with atypical anorexia nervosa who maintain a normal weight or even appear overweight. These individuals may exhibit significant disordered eating behaviors and psychological distress, yet their weight status can mask the presence of an eating disorder. As a result, they may go undetected and, consequently, remain undertreated.

This under-recognition of atypical anorexia nervosa in individuals with normal weight has significant implications for early identification and intervention. Healthcare professionals and clinicians should be aware that the presence of disordered eating behaviors and psychological distress should not be solely linked to weight status. Comprehensive assessment tools and diagnostic criteria that encompass the psychological and behavioral aspects of eating disorders are crucial in identifying and providing appropriate treatment for individuals who may not fit the stereotypical image of anorexia nervosa [16].

The association between obesity and eating disorders can be explained through proposed underlying mechanisms. It is suggested that specific psychological features, such as low self-esteem and poor body image, may be present in individuals with both obesity and eating disorders. These psychological factors can contribute to the development of obesity and the c-occurrence of disordered eating behaviors [17].

Understanding the interplay between obesity and eating disorders is crucial for comprehensive management and treatment approaches. By recognizing the psychological factors involved, healthcare professionals can provide targeted interventions to address these conditions' physical and psychological aspects.

SCOFF questionnaire proves to be a valuable and easily accessible screening tool, which can be utilized in the primary care setting to identify potential cases of eating disorders. The study’s findings highlight the possibility of uncovered or undetected cases of eating disorders in children and adolescents in the healthcare system. By incorporating the SCOFF questionnaire into routine assessments, healthcare professionals can increase their ability to identify individuals at risk of eating disorders and provide appropriate interventions.

Furthermore, the study reveals a significant relationship between a positive SCOFF questionnaire result and the risk of developing depression and early childhood obesity. This finding is consistent with a previous study that has linked the presence of eating disorders with higher risk of developing depression compared to individuals without eating disorders [18]. These findings underscore the importance of considering mental well-being as a critical determinant when addressing the issue of obesity in children and adolescents.

While this study did not specifically analyze other risk factors, such as bullying and teasing, existing literature has highlighted their relevance. Research has shown that individuals with eating disorders are more likely to have experienced bullying and teasing related to their appearance prior to the onset of the disorders [19]. These additional risk factors further emphasize the multifaceted nature of eating disorders and the need for comprehensive approaches that address both physical and psychological well-being.

In conclusion, the utilization of the SCOFF questionnaire as a screening tool helps uncover potential cases of eating disorders that may otherwise go undetected. The study's findings also highlight the significance of considering mental well-being, including the risk of depression and the impact of bullying and teasing, when addressing obesity and eating disorders in children and adolescents.

### Study limitation

The definition of obesity in children differs among the studies. Some studies used the Center for Disease Control's definition of obesity, and some others, such as this study, used the WHO definition of obesity in children. These differences in definition might lead to difficulty in comparing the results. Another limitation of the study is the use of the SCOFF questionnaire as the screening tool. Despite its brevity and ease of administration in primary care settings, in comparison with other tools, such as Eating Disorder Examination Questionnaire (EDE-Q), it is less sensitive and specific in detecting eating disorders [20]. Also, the SCOFF questions are more designed to screen for abnormal eating behavior for anorexia nervosa and bulimia. Unfortunately, it is less sensitive to the screening of other eating disorders such as avoidant/restrictive food intake disorder, binge eating disorder, other non-specified feeding or eating disorders, rumination disorder, and Pica [21].

## Conclusion and recommendation

This study marks an important step in addressing the prevalence of eating disorders among children and adolescents in the UAE, an area where there is limited data and inadequate implementation of early screening measures. The findings highlight the significance of obesity and eating disorders as pressing health concerns in our society.

As a recommendation, it is crucial to raise awareness among healthcare professionals, including physicians and school healthcare providers, about the importance of early screening for eating disorders in this population. By increasing knowledge and understanding, healthcare workers can play a pivotal role in early detection and intervention.

Additionally, there is a need for a comprehensive nationwide community screening program that encompasses not only eating disorders but also mental well-being and other high-risk behaviors among younger children and adolescents. Such a program would facilitate early identification and enable timely intervention and prevention strategies.

By prioritizing these recommendations and conducting further studies, we can improve the healthcare system’s response to eating disorders in children and adolescents, ultimately leading to better outcomes and enhanced overall well-being in this vulnerable population.

## Data Availability

The datasets generated during and/or analyzed during the current study are available from the corresponding author upon reasonable request.

## Abbreviations

UAE: United Arab Emirates
PHQ-2: Patient health questionnaire-2
CDC: Centers for disease control
BMI: Body mass index
WHO: World Health Organization
EDE-Q: Eating disorder examination questionnaire
